# A Smartphone App for Families With Preschool-Aged Children in a Public Nutrition Program: Prototype Development and Beta-Testing

**DOI:** 10.2196/mhealth.7477

**Published:** 2017-08-02

**Authors:** Pamela Hull, Janice S Emerson, Meghan E Quirk, Juan R Canedo, Jessica L Jones, Violetta Vylegzhanina, Douglas C Schmidt, Shelagh A Mulvaney, Bettina M Beech, Chiquita Briley, Calvin Harris, Baqar A Husaini

**Affiliations:** ^1^ Division of Epidemiology Department of Medicine Vanderbilt University Medical Center Nashville, TN United States; ^2^ Center for Prevention Research Tennessee State University Nashville, TN United States; ^3^ The National Academies of Sciences, Engineering, and Medicine Washington, DC United States; ^4^ Progreso Community Center Nashville, TN United States; ^5^ Meharry-Vanderbilt Alliance Meharry Medical College Nashville, TN United States; ^6^ Department of Electrical Engineering and Computer Science Vanderbilt University Nashville, TN United States; ^7^ School of Nursing Vanderbilt University Nashville, TN United States; ^8^ Departments of Pediatrics and Family Medicine University of Mississippi Medical Center Jackson, MS United States; ^9^ Department of Family and Consumer Sciences Tennessee State University Nashville, TN United States

**Keywords:** pediatric obesity, health education, public health informatics, mobile apps

## Abstract

**Background:**

The Special Supplemental Nutrition Program for Women, Infants, and Children (WIC) in the United States provides free supplemental food and nutrition education to low-income mothers and children under age 5 years. Childhood obesity prevalence is higher among preschool children in the WIC program compared to other children, and WIC improves dietary quality among low-income children. The Children Eating Well (CHEW) smartphone app was developed in English and Spanish for WIC-participating families with preschool-aged children as a home-based intervention to reinforce WIC nutrition education and help prevent childhood obesity.

**Objective:**

This paper describes the development and beta-testing of the CHEW smartphone app. The objective of beta-testing was to test the CHEW app prototype with target users, focusing on usage, usability, and perceived barriers and benefits of the app.

**Methods:**

The goals of the CHEW app were to make the WIC shopping experience easier, maximize WIC benefit redemption, and improve parent snack feeding practices. The CHEW app prototype consisted of WIC Shopping Tools, including a barcode scanner and calculator tools for the cash value voucher for purchasing fruits and vegetables, and nutrition education focused on healthy snacks and beverages, including a Yummy Snack Gallery and Healthy Snacking Tips. Mothers of 63 black and Hispanic WIC-participating children ages 2 to 4 years tested the CHEW app prototype for 3 months and completed follow-up interviews.

**Results:**

Study participants testing the app for 3 months used the app on average once a week for approximately 4 and a half minutes per session, although substantial variation was observed. Usage of specific features averaged at 1 to 2 times per month for shopping-related activities and 2 to 4 times per month for the snack gallery. Mothers classified as users rated the app’s WIC Shopping Tools relatively high on usability and benefits, although variation in scores and qualitative feedback highlighted several barriers that need to be addressed. The Yummy Snack Gallery and Healthy Snacking Tips scored higher on usability than benefits, suggesting that the nutrition education components may have been appealing but too limited in scope and exposure. Qualitative feedback from mothers classified as non-users pointed to several important barriers that could preclude some WIC participants from using the app at all.

**Conclusions:**

The prototype study successfully demonstrated the feasibility of using the CHEW app prototype with mothers of WIC-enrolled black and Hispanic preschool-aged children, with moderate levels of app usage and moderate to high usability and benefits. Future versions with enhanced shopping tools and expanded nutrition content should be implemented in WIC clinics to evaluate adoption and behavioral outcomes. This study adds to the growing body of research focused on the application of technology-based interventions in the WIC program to promote program retention and childhood obesity prevention.

## Introduction

The Special Supplemental Nutrition Program for Women, Infants, and Children (WIC) serves 9.3 million low-income, nutritionally at-risk mothers, infants, and children under age 5 years in the United States by providing free supplemental healthy foods, nutrition education, breastfeeding support, and health care referrals. Prevalence of childhood overweight and obesity has increased rapidly in recent decades, and risk for obesity increases sharply after age 5 years [1,2]. Prevalence is greater among black and Hispanic children compared to white children and greater among low-income families served by WIC compared to the general population [3-5]. The WIC program improves diet quality among low-income children [6], but participation in the program declines as children age, particularly among 2- to 4-year-olds [5].

Studies have found that the vast majority of WIC participants are satisfied with the nutrition education provided by WIC every 3 months, predominantly delivered in person in WIC clinics. However, repetition of information, long wait times, and accompanying children have been identified as barriers and predictors of program attrition [7,8]. Compared to the nutrition education component, WIC participants tend to have lower satisfaction with the process of redeeming their WIC benefits in the grocery store [8,9].

WIC benefits consist of approved food packages assigned to each WIC participant in the family, which list the specific types, brands, sizes, and quantities of products that can be purchased. In addition, a cash value voucher (CVV) provides a flat dollar amount per WIC participant that can be spent on fruits or vegetables, currently $8 for children and $10 for mothers. As of May 2017, the majority of states (30/50, 60%), including Tennessee, have not yet implemented electronic benefits transfer (EBT) statewide and continue to provide benefits in the form of paper vouchers; the majority of these remaining states are scheduled to transition to EBT in 2018 (including Tennessee) or 2019, with the deadline of 2020. Challenges with the WIC shopping experience can act as a barrier for participants to redeem all of their benefits and can lead to program attrition, particularly with paper vouchers [8-12]. An expert review panel recommended that in-clinic nutrition education be complemented with reinforcement education using innovative, multilevel strategies to make it easier for participants to purchase all of the nutritionally beneficial items included in their food packages and to consume them at home [13].

Very limited research has been conducted to date on the use of technology-based interventions in the WIC program [14-16]. Technology-supported interventions such as smartphone apps can impact a substantially larger number of people more frequently and usually at a lower cost than in-person education. Nearly two-thirds of all adults and 85% of young adults in the United States own a smartphone, with higher levels of ownership among racial/ethnic minorities compared to whites [17-19]. Mobile phone–based interventions have been effective with minority and low-income populations to improve nutrition and other health-promoting behaviors [20-23]. A handful of smartphone apps are commercially available that include some WIC shopping features and limited nutrition information or recipes. However, no published studies to date have reported on the development or testing of a smartphone app for WIC participants that combines nutrition education and WIC shopping tools.

This paper describes the development and beta-testing of the Children Eating Well (CHEW) smartphone app. This app was developed for WIC-participating families with preschool-aged children as a home-based intervention to reinforce WIC nutrition education and improve the WIC shopping experience. The objective of beta-testing was to test the CHEW app prototype with target users, focusing on usage, usability, and perceived barriers and benefits of the app.

## Methods

### Needs Identified by Stakeholders

As part of the larger Nashville CHEW for Health project, the CHEW Community Advisory Board (CAB) was formed in 2011, comprising 8 to 10 WIC participants and several nonprofit organizations focused on food security. The CAB provided input on the project’s objective to develop a home-based nutrition education intervention for WIC-participating families with preschool children ages 2 to 4 years with a particular focus on black and Hispanic families, given their elevated risk for obesity. CAB members indicated that they were largely satisfied with availability of nutritious foods available through the WIC program but would like for the cash value voucher for fruits and vegetables to be increased in value.

CAB members expressed that their main concern with the WIC program, similar to the findings of previous studies [8-11], was that the WIC shopping experience was confusing and included the following challenges: each family member having a different paper voucher; specific limits on brands, quantities, and sizes; allowed choices and combinations within categories; the CVV requiring math skills; embarrassment at the register when items did not match up to their approved benefits; and not always getting all of their allowed items due to these complexities. They strongly desired that something be done to make the process easier. In terms of specific topics for nutrition education, CAB members indicated that they would be interested in receiving quick and simple recipes with WIC items that their children would eat and practical advice on parent feeding strategies to help them more easily provide healthy food to their children.

The CHEW team also engaged in a series of conversations with state and local WIC program staff to identify program needs and feasible intervention strategies that would enhance the existing WIC program. WIC program staff expressed interest in opportunities to provide participants with tools and resources to complement the nutrition education they received during WIC clinic visits. The program staff had two main concerns: many participants not redeeming all of their monthly WIC benefits and lower program retention as children age past 2 years. They agreed with the feedback from the CAB that frustrations with the shopping experience could contribute to both of these problems. The combined feedback of the CAB and WIC program staff inspired the idea of creating an app with WIC shopping tools plus nutrition education to include simple recipes and practical advice.

In 2012, the team conducted the CHEW Nutrition Survey in a multiethnic sample of 150 families with WIC-participating children ages 2 to 4 years [24,25]. Analysis of the survey data on the dietary needs of the children informed the selection of dietary targets for the nutrition education to be developed. Compared to the US dietary guidelines that were in effect in 2012 [26], the children in the survey consumed less than the recommended amounts of vegetables, whole grains, dairy, and fiber [27]. Intake of whole grains and water was lowest among Hispanic children, while intake of fat, added sugars, and whole milk was highest among black non-Hispanic children, even though WIC did not provide whole milk to participants older than 2 years at the time. Commonly reported barriers to eating more fruits and vegetables included that the cost was more than the value of the CVV, parents or other family members did not like them, and parents did not have time to prepare fresh fruits and vegetables (unpublished findings). Finally, the team identified some cultural differences in food preferences for black and Hispanic children compared to white children, based on which fruits and vegetables the parents chose to purchase with the preschool child’s CVV.

### Theoretical Framework and Conceptual Model

**Figure 1:**
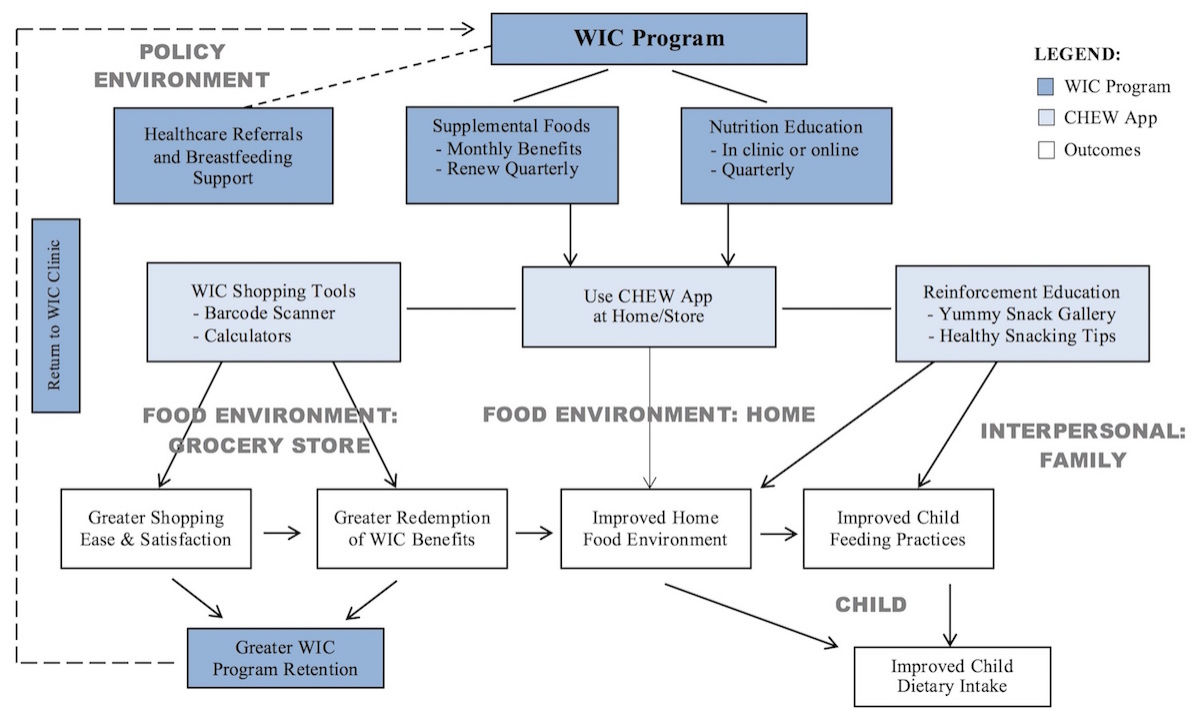
Conceptual model for Children Eating Well smartphone app based on socioecological framework.

Based on the input from the CAB and WIC program, we established the goal of developing a smartphone app for use as a home-based nutrition education intervention to complement and reinforce WIC’s in-clinic nutrition education with a specific focus on black and Hispanic children ages 2 to 4 years. The theoretical framework that guided the development of the CHEW app was the socioecological model [28-30]. The CHEW app interplays with the following 5 levels of influence on child dietary intake (Figure 1):

1. The *policy level* represents the state WIC program that determines the approved food packages compliant with federal regulations for each family and provides periodic nutrition education and other services.
2. The *grocery store food environment* is where clients will use the app to facilitate WIC shopping and ideally maximize purchasing all of their eligible items.
3. The *home food environment* is where the user will interact with the app most of the time, bringing home more healthy food, planning shopping, and viewing content.
4. The *interpersonal level* is where the parent will interact with the child, implementing tips learned through the app for feeding the child. Parents are the primary agents of change within the family, especially for younger children; thus they are the primary audience for the app.
5. The *individual level* represents the child, where all of the influences will converge to impact the child’s consumption of food and beverages.

The concept is for the app to improve the WIC shopping experience, which should lead to maximizing benefit redemption and an improved home environment as well as increased satisfaction and program retention. Moreover, the nutrition education provided through the app will improve the home food environment and improve parent-feeding practices, which will lead to improved child dietary intake.

### Development of the Children Eating Well Smartphone App Prototype

#### Overview

**Figure 2:**
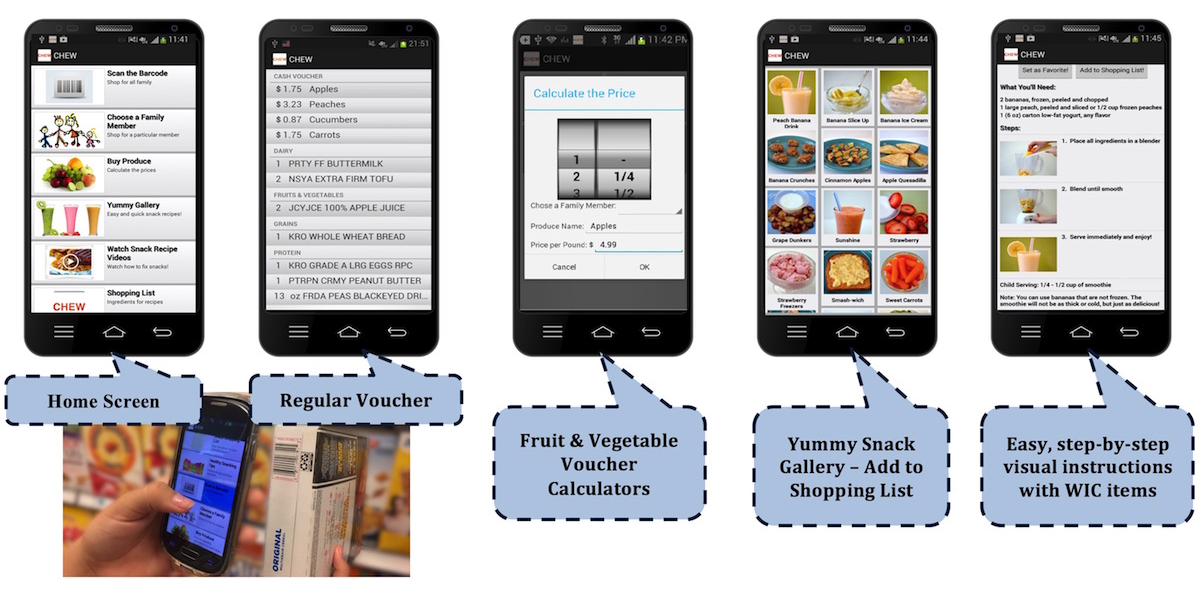
Screenshots and features of Children Eating Well smartphone app prototype.

The CHEW team carried out an iterative [31], user-centered [32] design process to develop the CHEW app prototype with periodic input on concepts, content, and functional prototypes from the CAB and the WIC program as the target end-users. Computer science engineering graduate and undergraduate students, under supervision of engineering faculty, programmed the prototype version for the Android operating system. The goals of the app are to improve the WIC shopping experience, increase benefit redemption, and improve parent snack feeding practices. The app consists of 2 components: WIC shopping tools to make the WIC shopping process easier and nutrition education for parents of 2- to 4-year-olds [33,34]. See screenshots in Figure 2. All app content was prepared in both English and Spanish with the assistance of one of our key partners, a Hispanic community organization. Users have the option to switch between English and Spanish versions of the app.

#### WIC Shopping Tools

Several app features were developed to assist users with making selections while in the grocery store. Two large grocery chains where the majority of WIC benefits are redeemed in Tennessee provided the research team with lists of all the WIC-authorized items carried by each store and the corresponding universal product codes (UPC). The research team combined the databases and coded approximately 1000 items to indicate to which regular vouchers each corresponded as an allowed item along with over 12,000 fruit and vegetable items that could be purchased with CVVs. In addition, the programmers constructed logical arguments to represent which items, combinations of items, and item limits were included on each regular voucher type.

For the prototype version, only standard food package types were included, excluding food packages for special dietary needs. The programmers built a barcode scanner feature that enables the user to scan the UPC barcode of any item in the store to see if the item is (1) approved by WIC and (2) on one of the family members’ vouchers. Users can then select if they want to purchase the item, for which voucher, and the quantity, if applicable. The app notifies users when they reach the limits for specific categories. At any time during the shopping trip, users can view the products and quantities allowed for each voucher and a running list of the items they have selected for each voucher.

For the CVV, the programmers built calculator tools to assist the shopper with using the specified dollar amount for each CVV. The cost of fruits and vegetables is challenging for participants to keep track of given the various ways that they are priced in grocery stores. Thus, 4 calculators are available depending on which type of fruits or vegetables the user wants to purchase: packaged fresh—fixed price (eg, bag of carrots, package of strawberries), packaged frozen—fixed price (eg, box of frozen spinach, bag of frozen peaches), loose produce—price per pound (eg, apples or green beans priced per pound), and loose produce—price per item (eg, individual cucumbers or watermelons priced per unit). Since prices in each store vary day to day, the app does not include a price database, so the user is prompted to enter the package or unit price and quantity. The cost for the total quantity is then calculated. The app keeps a running tab of the amount spent on each CVV and alerts the user when maximum values are reached.

#### Nutrition Education

Based on feedback from the CAB and WIC program and the survey findings for the prototype version, the team chose to focus the nutrition education content on age-appropriate snacks and beverages for preschoolers. The two nutrition education features in the prototype version are a Yummy Snack Gallery and Healthy Snacking Tips. The objective of these features is to give parents practical skills and strategies to improve the following dietary targets for children: increased intake of fruits, vegetables, fiber, low-fat milk, and water and decreased intake of sugar-sweetened beverages.

In the prototype version, the Yummy Snack Gallery includes 35 snack and beverage recipes developed by the CHEW team to be quick and easy to prepare, have few ingredients, include WIC-approved items, require no cooking skills, and be appealing to 2- to 4-year-olds. Each recipe includes images for visual step-by-step instructions. The recipes were selected to target the dietary preferences of black and Hispanic WIC participants based on findings from the CHEW Nutrition Survey of the most frequent fruits and vegetables purchased by each group. Examples of recipes include fast collard greens, peanut butter and fruit smash-wich, avocado toast, chayote salad, veggie boats, and peachy banana smoothie. Users can browse the snack gallery, view the details of each recipe, flag specific recipes as favorites, and add the ingredients to a snack shopping list.

In the prototype version, the Healthy Snacking Tips feature includes a database of 21 short educational messages and 21 reminders to use the various app features. The educational messages are based on evidence from the scientific literature about parent feeding strategies to provide parents with advice on practical strategies they can employ with their preschool children to improve the targeted dietary behaviors. The main topic areas cover beverages (eg, “Whole milk is for toddlers under two. For older kids and adults, gradually switch to 2% then 1% and then skim. Try mixing them for an easier switch!”), kid-friendly snacking (eg, “Let healthy foods fill the gaps! If your child misses fruit at lunch, offer it as an afternoon snack.”), introducing new foods (eg, “It may take more than 12 tries for your child to like a new food. Stay positive and keep motivating them to try a bite!”), parents as role models (eg, “Be a great role model! Let your child see you enjoying fruits, vegetables, water, and low-fat dairy every day.”), and health benefits (eg, “Fiber helps the tummy do its work and helps you go regularly! Get it from: Apples * Carrots * Broccoli * Pears * Spinach”). These topics were selected with input from the CHEW CAB. Examples of reminders include, “Remember to use your WIC shopping tools when you're ready to buy WIC items at the store,” and “Just a few minutes until snack time? Check out the Yummy Snack Gallery for quick and tasty ideas!”

The app is programmed to make the messages appear on the phone via push notifications per a predetermined schedule. In the prototype version, the schedule was set to vary the number of notifications sent each week, with each messages being sent twice across the 3-month testing period. The default time to send the notification is 10:00 AM on the designated day, and the user has the option to change the time.

### Study Sample and Recruitment

The prototype app was beta-tested with a sample of 80 mothers of WIC-participating children ages 2 to 4 years, stratified by race/ethnicity of the child with 40 black non-Hispanic children and 40 Hispanic children of any race. Study participants were enrolled from August 2014 through January 2016. To recruit families actively enrolled in the WIC program, potentially eligible families were randomly sampled from periodic lists of black and Hispanic participants 2 to 4.5 years of age who made a WIC clinic visit in the previous 3 months. The upper age cutoff was set at 4.5 (4 years and 6 months) to allow time to recruit families and complete the testing period before the child turned 5 and would no longer be eligible for WIC benefits. The study team then contacted the adult contact person to invite the mother to be screened and participate in the study if eligible. The mother was targeted for inclusion in the study since mothers are typically the parent primarily responsible for feeding young children in families. They were first contacted by mail and given the opportunity to opt out of further contact, then study staff followed up with phone calls.

Eligibility screening was completed over the phone in English or Spanish, per the preference of the respondent. Inclusion criteria were as follows: child is between 2 and 4.5 years of age, either non-Hispanic black/African American or Hispanic of any race (as identified by a parent or guardian), currently receives benefits from WIC, will receive benefits for the 3 months following enrollment, and receives one of the standard food packages (not special dietary needs); mother is 18 years of age or older (or if under 18 years of age, her guardian provides consent to participate), currently owns and regularly uses an Android smartphone, and uses the family’s WIC vouchers at one of the two major grocery chain stores included in the UPC database. The following exclusion criteria were applied: mother does not speak and read English or Spanish, is currently pregnant, currently has a child under the age of 6 months, is unable to provide consent, or someone other than the mother regularly does the WIC shopping for the child participant or family. The study inclusion and exclusion criteria were extensive and strict in order to help ensure that we enrolled people who would most likely be able to use the app and complete the study; it was not intended as a model for how the app will be disseminated in practice settings in the future.

Out of all the families on the sampled lists, 22% were not reachable due to incorrect contact information, 20% were nonresponsive, 10% opted out of further contact or declined to be screened, and the remaining 48% were successfully screened for eligibility. Among those screened, approximately two-thirds (69%) did not qualify based on the inclusion and exclusion criteria listed above. The most common reasons for failing screening were as follows: not owning an Android smartphone (39%), having an infant under 6 months of age (15%), not shopping regularly at one of the two targeted chain grocery stores (14%), currently being pregnant (10%), and speaking a language other than English or Spanish (7%). Potential participants may have owned smartphones with other operating systems (eg, iPhone iOS, Windows), but for the purposes of testing the prototype programmed on the Android platform, they were not eligible to participate in this study. Among those screened, 14% were deemed eligible but did not follow through with the baseline interview appointment or subsequently declined and 17% were deemed eligible and enrolled in the study (ie, 55% of those who were screened as eligible decided to enroll). This study was approved by the Institutional Review Board of the lead institution.

### Study Design and Procedures

Beta-testing of the CHEW app prototype was conducted using an observational design based on data collected after the testing period. Interested mothers who were screened as eligible scheduled an in-home visit with trained interviewers to complete enrollment. After completing the informed consent process, enrolled mothers completed a baseline questionnaire in English or Spanish. The interviewers then installed the CHEW app prototype on their Android smartphones, loaded the family’s current WIC voucher information into the app, gave the mother an overview of how to use the app, and asked her to test the app during the next 3 months. The app was successfully installed for 74 of the 80 enrolled mothers (92%); partially installed for 3/80 mothers (4%), meaning that the main app worked, but the scanning function was not fully operable; and was not successfully installed for 3/80 mothers (4%) due to incompatibility of their model of Android phone.

A second in-home visit was scheduled for 3 months after the baseline visit, during which the mothers completed a follow-up questionnaire that included a series of questions about their experiences with the app prototype. At the follow-up visit, the interviewers also attempted to manually download an app usage log from the participant’s phone that tracked the frequency of using specific features in the app. Follow-up visits were successfully completed with 63/80 mothers (79% retention), with 7/80 (9%) declining follow-up and 10/80 (13%) lost to follow-up. Among the 63 who completed the follow-up visit, usage logs were successfully downloaded from 42/63 participants’ phones (67%). Interviewers were unable download logs from the remaining participants: 3/63 (5%) whose installation failed at baseline due to phone incompatibility, 11/63 (18%) who had replaced their phones since the first interview and no longer had the app, 3/63 (5%) whose phone was currently broken or lost, and 4/63 (6%) in which the interviewer was unable to locate and retrieve the log file from the phone. Participants received a $25 for each completed in-home visit.

### Measures

#### App Usage Log

##### Overview

The app was programmed with a simple logging function to record time-stamped notations when certain activities or events were performed by the user in the app, such as opening the app or one of the features, into an archived file. The log could then be manually downloaded from the phone in the form of a raw text file. Next our team converted each text file into a spreadsheet with usable data fields. Then we aggregated all of the event rows up to the level of the individual user, creating computed variables using arithmetic functions such as sum or mean. Finally the data points for each user were merged into a cumulative log database with one row per participant.

##### Frequency and Duration of App Sessions

Individual app events were sorted by date and time to identify discrete user sessions. A new session was operationalized as any new event (eg, opening the home page) occurring at least one hour after the previous event. When aggregating the data to the individual level, we hand-counted the number of discrete app sessions per user. At the same time, we calculated the duration of each session in minutes, then upon aggregation calculated the average duration across all of the individual user’s sessions.

##### Frequency of Using App Features

Within each participant’s usage log, we counted the frequency of several specific logged events as indicators for using a specific app feature. For using the WIC shopping tools, we counted the number of times the user selected a grocery store (one of the two stores included in the app) to initiate a shopping session as well as the number of times the user opened any of the produce calculators. For use of the snack gallery, we counted the number of times the user opened one of the snack recipes. It was not possible to track receipt or reading of the healthy snacking tips messages using the log since these messages were delivered on a predefined schedule to the user through the Android phone’s native push notification function.

#### Questionnaire

At baseline, mothers provided sociodemographic information about themselves, the preschool-aged child, and the family. At follow-up, mothers responded to a series of items about their experiences with the CHEW app prototype during the testing period, described below.

##### Self-Reported Usage

The mother reported whether or not she had used each of the main features of the app at least once (Yes or No) during the past 3 months: WIC shopping tools, snack gallery, and healthy snacking tips. A “yes” response on any of these 3 items was then coded in a new variable to indicate using any of the features in the past 3 months.

##### Usability

Concepts from an existing smartphone app usability model [35] were used to construct a series of usability items specific to the features of the CHEW app in terms of ease of use, helpfulness, usefulness, and satisfaction. The response choices for all of the items consisted of 5-point Likert scales ranging from 1=strongly disagree to 5=strongly agree, with higher values indicating greater usability. Some examples of items include, “The Snack Gallery recipes were easy to follow” (ease of use), “The fruit and vegetable calculator was helpful” (helpfulness), “The Healthy Snacking Tips gave me new information” (usefulness), and “I would recommend the WIC Shopping Tool to other WIC families” (satisfaction).

##### Perceived Benefits

A series of items was created specific to the CHEW app prototype to assess the extent to which the user felt the app helped the mother perform the targeted behaviors. For each app component (shopping tools, snack gallery, snacking tips), the items asked whether this component helped the user perform specific WIC shopping tasks or recommended parent feeding strategies. The response choices for all of the items consisted of a 5-point Likert scale ranging from 1=strongly disagree to 5=strongly agree, with higher values indicating greater benefits. Examples of items include, “The WIC Shopping Tool made checking out easier with my cash value vouchers for fruits and vegetables,” “The Snack Gallery helped me introduce new vegetables to my child,” “The Healthy Snacking Tips helped me give my child water instead of sugary drinks,” and “The Healthy Snacking Tips helped me with my picky eater.”

##### Qualitative User Feedback

In addition to the structured, quantitative questionnaire items described above, the interviewers asked the participants several open-ended questions to elicit qualitative responses of user feedback regarding the key app features. For all of these questions, the mother could answer however she wanted, and the interviewer typed her response into the data collection form as close to verbatim as possible.

Reasons for nonusers: If the participant indicated in the usage questions above that she did not use or view one of the 3 components (shopping tools, snack gallery, healthy snacking tips) at all during the past 3 months, the interviewer asked why she did not use the feature, as an open-ended question.

Barriers and benefits for users: At the end of each set of usability and benefits items for each of the 3 main app features, the interviewer asked the following 2 open-ended questions regarding each feature: “Is there anything you would like to see changed about the [fill in] feature in the CHEW app?” and “Do you have anything else you'd like to say about the [fill in] feature in the CHEW app?”

### Data Analysis

Analyses were performed on the data from the 63 mothers who completed the posttesting follow-up questionnaires and the subset of 42 mothers with app usage logs that were downloaded at follow-up. Sample characteristics were described using cross-tabulations with chi-square tests to indicate associations with race/ethnicity of the child, since the sample was stratified by African Americans and Hispanics. Descriptive statistics were used to report the other quantitative variables, using frequencies and percentages for categorical variables and means with standard deviations for continuous and ordinal variables.

The qualitative responses to the open-ended questions were pooled together, reviewed to identify emergent common themes where applicable, and assigned categories corresponding to the themes. The themes were then summarized in a table with counts to provide a general idea of how often they were mentioned by participants. The responses of nonusers for each feature (ie, reasons for not using a feature at all) were summarized separately from the responses of mothers who had at least some experience using a feature. For these mothers, the themes emerging from the 2 open-ended questions were divided into barriers and benefits for using the respective app feature.

For purposes of informing development of future versions of the app, the team chose to interpret mean scores of 4.0 or higher as successful in usability or benefits, mean scores of 3.0 to 3.99 as a moderately positive responses that indicate areas needing focus for improvements in usability or benefits, and mean scores below 3.0 as not meeting minimum criteria for usability or benefits and needing major changes.

## Results

### Sample Characteristics

Table 1 reports the characteristics of the sample by racial/ethnic group of the child. The racial/ethnic group of the mother matched that of the child for all but 2 women, one in each group (results not shown). The majority of children were 2 or 3 years old, given that the upper age cut off was 4.5 years. The sample had slightly more male than female children, all of whom were born in the United States. For mothers, the largest age group was 25 to 34 years. Roughly equal percentages of mothers were either married or single/never married. All but one of the mothers of Hispanic children were born outside of the United States. Over half of the mothers had only one child receiving WIC, approximately one-third had 2 family members receiving WIC, and nearly one-tenth had three or more family members. More than 4 out of 5 families also received benefits from the federal Supplemental Nutrition Assistance Program (SNAP) in addition to WIC. Race/ethnicity of the child was significantly associated with mothers’ education (*P*<.001), employment status (*P*=.01), country of birth (*P*<.001), and food insecurity (*P*=.001). We compared the baseline demographic characteristics in Table 1 for those who did and did not complete the 3-month follow-up interview. The only significant difference was that families also receiving SNAP benefits were more likely to complete follow-up (results not shown).

**Table 1:** Sample characteristics from the Children Eating Well smartphone app prototype study (note: some column percentages do not add up to 100% due to rounding).

Variable		Black children N=33 n (%)	Hispanic children N=30 n (%)	*P* value
**Child—age**				
	2 years	18 (54)	13 (45)	.09
	3 years	14 (42)	10 (35)	
	4 years	1 (3)	6 (21)	
**Child—gender**				
	Male	17 (52)	17 (57)	.68
	Female	16 (49)	13 (43)	
**Child born in United States**				
	No	0	0	1.00
	Yes	33 (100)	30 (100)	
**Mother—age**				
	18-24 years	5 (17)	1 (3)	.21
	25-34 years	14 (47)	18 (60)	
	35-44 years	11 (37)	11 (37)	
**Mother—marital status**				
	Married	10 (30)	17 (57)	.10
	Single, never married	20 (61)	12 (40)	
	Single, divorced	3 (9)	1 (3)	
**Mother—education**				
	<High school degree	2 (6)	15 (52)	<.001
	High school degree	8 (24)	10 (35)	
	Any college/technical	22 (67)	4 (13)	
**Mother—employment**				
	Not employed	12 (36)	22 (73)	.01
	Employed full-time	14 (42)	5 (17)	
	Employed part-time	7 (21)	3 (10)	
**Mother born in United States**				
	No	4 (12)	29 (97)	<.001
	Yes	29 (88)	1 (3)	
**Family members on WIC^a^**				
	1	19 (57)	18 (60)	.73
	2	11 (33)	9 (30)	
	3 or more	3 (9)	2 (9)	
**Family receives SNAP^b^**				
	No	4 (12)	5 (17)	.61
	Yes	29 (88)	25 (83)	
**Run out of money for food**				
	Never	9 (27)	1 (3)	.001
	Sometimes	11 (33)	5 (17)	
	Often	2 (6)	12 (41)	
	Nearly every month	11 (33)	11 (38)	

^a^WIC: Special Supplemental Nutrition Program for Women, Infants, and Children.

^b^SNAP: Supplemental Nutrition Assistance Program.

### Usage

Table 2 presents usage of the main app components. According to the available app logs, on average mothers engaged in 12.57 discrete sessions, which roughly translates to once a week over a 3-month period. More than one-third of mothers (15/42, 36%) used it more than 12 times, or more than once a week. However, frequency of usage varied substantially, with a range from 0 to 70 and fairly even distribution across the various categories. Over 20% (9/42) only used the app once or not at all, while over half (22/42, 52%) used it more than 6 times, or more than twice a month. The average duration of sessions also varied substantially, with a mean of 4.67 minutes and a range from 0 to 22 minutes, which was spread fairly evenly across 2-minute intervals up to 6+ minutes.

Mothers engaged in a shopping session in the app by selecting a grocery store on average 5.14 times, with a range of 0 to 19. Over half (22/42, 52%) selected a store more than 3 times, or at least once a month. Users opened the produce calculators on average 3.21 times, or approximately once a month, ranging from 0 to 17 times. Over one-third opened the calculator more often than 3 times (15/42, 36%). The snack gallery had the highest average usage at 12.76 times, or roughly once a week. However, the wide range of 0 to 111 times and SD 24.67 suggest a right-tail skewed distribution influenced by a few outliers. Nevertheless, 40% (17/42) of participants opened a recipe 6 or more times, or more than twice a month. Each feature had no logged record of use for nearly a third of mothers.

In the self-reported questionnaire items, 4 out of 5 mothers (51/63, 81%) reported that they used the WIC shopping tools, over two-thirds (43/63, 68%) said they used the snack gallery, and three-fourths (47/63, 75%) indicated that they received or read the healthy snacking tips. Combined, 9 out of 10 mothers (57/63, 91%) reported using at least 1 of the 3 components at some point during the 3 months. This overall percentage coincides with the app log data indicating that 90% (38/42) opened at least 1 app session, and the 2 estimates for using the snack gallery at least once were similar. However, mothers self-reported using the shopping tools at least once more often than the logged count of users selecting a store or using the produce calculators at least once.

### Qualitative Feedback on Barriers and Benefits

Table 3 includes a summary of the reasons given by mothers who indicated they had not used a specific app feature at all during the 3-month test period. Many in this group experienced technical barriers that precluded their ability to use a specific feature or the entire app, such as a broken phone, unsuccessful installation, and problems with features working properly on their phone. A few pointed to the app not being easy enough to use, lack of interest in the content, forgetting to use it, or not noticing alerts.

Also included in the table are themes that represent barriers and benefits mentioned by mothers in the open-ended questions. Notably, the overwhelming majority of users did not mention any barriers to using each of the features when asked what they would want to change about them. Nevertheless, the users identified some important issues with the shopping tools that may have interfered with some users taking full advantage of them. The most common problem was the barcode scanner not functioning well on certain Android phones. Some noticed potential errors in the database of WIC-approved items provided by the 2 grocery store chains as well as a delay in our team updating the database when WIC implemented some food package changes in October 2014. A few users experienced some challenges in using the shopping tools. At the same time, even though the open-ended questions did not specifically ask mothers to comment on what they liked about the app, many were enthusiastic about how they liked the shopping tools and found them to be helpful.

Many mothers also expressed enthusiasm and satisfaction with the snack gallery, saying they liked it, it was helpful, their children loved it, and it was easy and affordable. The common barrier noted for the snack gallery was a demand for it to be expanded with more recipes. One mother also said she would prefer to use it on a different device besides her phone.

Regarding the healthy snacking tips, a couple users experienced technical issues in receiving the alerts, some did not like the delivery schedule, and some were not interested in the information or reminders. Roughly as many mothers pointed out that they liked the tips or wanted more. A few said they were helpful, specifically for helping them buy or eat more fruits and vegetables.

**Table 2:** Children Eating Well smartphone app prototype usage (note: some column percentages do not add up to 100% due to rounding).

Variable			n (%)
**Logged app usage (n=42)**			
	**App sessions (mean 12.57, SD 14.98)**		
		None	4 (9.5)
		1	5 (11.9)
		2-3	6 (14.3)
		4-6	5 (11.9)
		7-9	4 (9.5)
		10-12	3 (7.1)
		13-15	1 (2.4)
		16-18	4 (9.5)
		19 or more	10 (23.8)
	**Average minutes per session (mean 4.67, SD 4.29)**		
		Less than 2	11 (28.9)
		2-3.9	8 (21.1)
		4-5.9	8 (21.1)
		6 or more	11 (28.9)
	**Times selected store for shopping (mean 5.14, SD 5.43)**		
		None	13 (31.0)
		1-3	7 (16.7)
		4-6	10 (23.8)
		7-9	2 (4.8)
		10-12	4 (9.5)
		13 or more	6 (14.2)
	**Times opened produce calculators (mean 3.21, SD 4.25)**		
		None	14 (33.3)
		1-3	13 (31.0)
		4-6	8 (19.0)
		7-9	4 (9.5)
		10 or more	3 (7.1)
	**Times opened a snack recipe (mean 12.76, SD 24.67)**		
		None	12 (28.6)
		1-5	13 (31.0)
		6-10	5 (11.9)
		11-20	4 (9.5)
		21-30	3 (7.1)
		31 or more	5 (11.9)
**Self-reported usage in follow-up interview (n=63)**			
	Used any feature at least once		57 (90.5)
	Used WIC^a^shopping tools at least once		51 (81.0)
	Used snack gallery at least once		43 (68.3)
	Viewed healthy snacking tip alerts at least once		47 (74.6)

^a^WIC: Special Supplemental Nutrition Program for Women, Infants, and Children.

### Usability and Perceived Benefits

Table 4 reports means and SD for the usability and perceived benefits items for each of the app prototype components. None of the items scored below a 3 on usability or perceived benefits dimensions, which would have indicated a need for major changes.

For usability, the WIC shopping tools overall, the barcode scanner, and produce calculators scored just under 4 for ease of use (3.81-3.98), indicating a need for some improvement. The barcode scanner also scored just under 4 for helpfulness (3.90) and usefulness/correct information (3.96), while the calculators and overall tools scored above 4 on these dimensions (4.16-4.40). The WIC shopping tools scored above 4 (4.33) on satisfaction in terms of willingness to recommend them to other WIC participants. In terms of perceived benefits of the shopping tools, 6 of the 7 items scored above 4 (4.10 to 4.51), with 1 item scoring 3.57 for the tools helping to spend more of the CVV, indicating a need for some improvement to facilitate that target behavior.

Mothers who used the Yummy Snack Gallery in the previous 3 months scored it high on all of the indicators of ease of use, helpfulness, usefulness, and satisfaction, with averages ranging from 4.63 to 4.95. For the 2 perceived benefits of the Yummy Snack Gallery, introducing new fruits scored just under 4 (3.93) and introducing new vegetables scored just above 4 (4.05).

Among mothers who viewed the Healthy Snacking Tips in the previous 3 months, on average they also scored it above 4 on all of the indicators, ranging from 4.34 to 4.68. For perceived benefits, 6 of the 8 items scored below 4, ranging from 3.20 to 3.96, with the lowest for switching to low-fat/skim milk. The 2 items scoring above 4 were reducing sugary drinks and offering more fruits.

## Discussion

### Principal Findings

Overall, our beta-testing successfully demonstrated the feasibility of using the CHEW app prototype with mothers of WIC-enrolled black and Hispanic preschool-aged children. Study participants testing the app for 3 months used the app on average once a week for approximately 4 and a half minutes per session, according to app usage logs. However, substantial variation was observed, with very low to no use among 20% of mothers while another half of mothers used it more than twice a month. Usage of specific features averaged at 1 to 2 times per month for shopping-related activities and 2 to 4 times per month for the snack gallery. Over two-thirds of users were tracked using the shopping tools or the snack gallery at least once over the 3-month period. This moderate level of engagement with the prototype app suggests that the app generated enough interest and perceived benefits for most participants to try it and many to continue using it periodically. In order to further expand initial uptake and ongoing engagement with the next version of the app, it will be crucial to maintain high usability and appeal to generate perceived benefits among users.

Mothers classified as users rated the app’s WIC Shopping Tools relatively high on usability and benefits, although variation in scores and qualitative feedback highlighted several barriers that need to be addressed: improving the functionality of the barcode scanner, making the shopping tools and produce calculators easier to use, and establishing a process for resolving occasional errors or changes in the database of WIC-approved items. Explicit attention should be paid to planning a mechanism for a future version of the app to communicate seamlessly with WIC’s new EBT system in the future.

The Yummy Snack Gallery and Healthy Snacking Tips scored higher on usability than benefits, suggesting that the nutrition education components may have been appealing but too limited in scope and exposure. Qualitative feedback revealed that some users had technical problems with push notifications, some wanted access to more recipes and tips, and individual preferences varied regarding the timing and frequency of Healthy Snacking Tips. In addition to expanding the quantity and frequency of nutrition content, users may benefit from individual tailoring of content to enhance perceived benefits [36,37].

**Table 3:** Summary of qualitative feedback from users on Children Eating Well smartphone app prototype features.

Feature	Response	Reason	Number
**WIC^a^Shopping Tools**			
	**Reasons for nonusers (n=12)^b^**		
		Lost/broken phone	4
		Initial installation failed	3
		Problems with scanner	3
		Inconvenient	1
		Confusing	1
		Hard in store with children	1
	**Barriers for users (n=51)^c^**		
		None	34
		Scanner slow/inconsistent	8
		Incorrect after WIC change	3
		Items say non-WIC approved	3
		Confusing	3
		Needed more instructions	2
		Forgot to use	1
		Limited time to shop	1
		Would like to include prices	1
	**Benefits for users (n=51)**		
		Liked it/great/worked well	4
		Shopping tools were helpful	2
		Scanner was helpful	1
		Helpful for using CVV^d^	1
		Should give to everyone	1
**Yummy Snack Gallery**			
	**Reasons for nonusers (n=20)**		
		Forgot to use	5
		Lost/broken phone	3
		Unsuccessful installation	3
		No reason given	3
		Problems with other features	2
		Not interested in recipes	2
		Limited variety in recipes	1
		Did not know how to use	1
	**Barriers for users (n=43)**		
		None	33
		Wanted more recipes	9
		Wanted on another device	1
	**Benefits for users (n=43)**		
		Liked it/great/fun	6
		Helpful	5
		Kids loved it	1
		Easy	1
		Affordable	1
		Good for everyone (non-WIC)	1
**Healthy Snacking Tips**			
	**Reasons for nonusers (n=16)**		
		No reason given	8
		Unsuccessful installation	3
		Lost/broken phone	3
		Did not notice the alerts	2
	**Barriers for users (n=47)**		
		None	38
		Stopped receiving alerts	2
		Messages too frequent	2
		Child already ate healthy	2
		Not interested in reminders	1
		Repeated messages	1
		Time of message delivery	1
	**Benefits for users (n=43)**		
		Great info/loved it	3
		Helpful to buy/eat more F/V^e^	2
		Wanted more tips	1
		Reminders were helpful	1
		Worked well	1

^a^WIC: Special Supplemental Nutrition Program for Women, Infants, and Children.

^b^One person provided 2 reasons.

^c^Five people provided 2 reasons.

^d^CVV: cash value voucher.

^e^F/V: fruits and vegetables.

**Table 4:** Usability and perceived benefits of Children Eating Well smartphone app prototype features (note: response choices for each item ranged from 1=strongly disagree to 5=strongly agree. Interpretation: successful—4.0 and higher; needs improvement—3.0 to 3.99; needs major changes—below 3.0).

Feature				Mean (SD)
**WIC^a^Shopping Tools (n=51)**				
	**Usability**			
		**Ease of use**		
			Overall	3.98 (1.12)
			Barcode scanner	3.81 (1.32)
			Produce calculators	3.95 (1.16)
		**Helpful**		
			Overall	4.40 (0.90)
			Barcode scanner	3.90 (1.42)
			Produce calculators	4.16 (1.00)
		**Useful: correct information**		
			Overall	4.29 (1.15)
			Barcode scanner	3.96 (1.52)
			Produce calculators	4.39 (0.97)
			Satisfaction: recommend to others	4.33 (1.05)
	**Perceived Benefits**			
		Keep track of family vouchers		4.45 (1.08)
		WIC shopping easier		4.10 (1.13)
		Show foods allowed to buy		4.51 (0.83)
		Regular checkout easier		4.10 (1.14)
		CVV^b^checkout easier		4.30 (1.09)
		Buy all regular voucher items		4.37 (1.06)
		Spend more of CVV		3.57 (1.35)
**Yummy Snack Gallery (n=43)**				
	**Usability**			
		Ease of use: simple		4.81 (0.50)
		Ease of use: easy to follow		4.84 (0.43)
		Helpful: photos and steps		4.95 (0.21)
		Useful: age appropriate		4.77 (0.43)
		Useful: fruits that I buy		4.88 (0.39)
		Useful: vegetables that I buy		4.63 (0.73)
		Satisfaction: recommend to others		4.84 (0.37)
	**Perceived benefits**			
		Introduce new fruits		3.93 (1.24)
		Introduce new vegetables		4.05 (1.17)
**Healthy Snacking Tips (n=47)**				
	**Usability**			
		Helpful		4.68 (0.59)
		Useful: new information		4.40 (0.83)
		Satisfaction: enjoy receiving		4.34 (0.94)
		Satisfaction: recommend to others		4.60 (0.77)
	**Perceived benefits**			
		Increase child's water		3.77 (1.32)
		Reduce sugary drinks		4.07 (1.29)
		Replace sugary drinks with water		3.96 (1.35)
		Switch to low-fat/skim milk		3.20 (1.58)
		Offer more fruits		4.11 (1.12)
		Offer more vegetables		3.94 (1.24)
		Help with picky eater		3.68 (1.31)
		Improve my own diet		3.98 (1.27)

^a^WIC: Special Supplemental Nutrition Program for Women, Infants, and Children.

^b^CVV: cash value voucher.

Qualitative feedback from mothers classified as nonusers pointed to several fundamental barriers that could preclude some WIC participants from using the app at all. These included technical problems with their phone (eg, lost or broken), the app not working properly on their phone, challenges in understanding how to use the app, forgetting that the app is on their phone, and lack of interest. One challenge of programming apps on the Android platform is variation in the compatibility of the app across the multiple manufacturers of Android smartphones to use features such as a barcode scanner and push notifications. The multiple manufacturer situation does not apply to the iOS platform, so it my have fewer compatibility issues in the future.

This study adds to the small but growing body of research focused on the application of technology-based interventions in the WIC program to promote program retention and healthy weight gain during childhood. A report on WIC research agenda recommendations identified the need for interventions to increase benefit redemption and consumption of WIC foods, effective approaches to reinforce WIC education between quarterly visits, and effective use of technology in WIC nutrition education [38]. A study in New York found that the two most common reasons for participants to drop out of WIC were negative shopping experiences and low perceived value of the WIC food package [39]. The CHEW app addresses these needs as a technology-based reinforcement intervention designed to increase benefit redemption through WIC shopping tools and consumption of WIC foods through built-in nutrition education tools. In addition, the app could potentially have a positive effect on retention in the WIC program in preschool ages by improving the shopping experience for parents and caregivers and providing greater incentive to remaining in the program.

A study in Michigan found that online WIC education modules were as effective as traditional WIC education for increasing fruit and vegetable consumption [14]. In a 2011 survey of WIC clients/parents in western US states, the majority among ages 20 to 31 years reported interest in the following options for WIC-related services: checking WIC EBT balance online, accessing WIC-authorized food guides online, a smartphone UPC barcode scanning app to check WIC-authorized items, and online recipes [15]. In a recent focus group study, WIC participants expressed interest in the idea of receiving a comprehensive WIC app with multiple features such as recipes, shopping lists, how-to videos, and tools for tracking health behaviors [16].

### Limitations

As a common limitation among most studies using self-report data, the survey responses of participants could have been influenced by social desirability bias [40]. Thus, we complemented the subjective self-report data with objective app usage logs to confirm patterns of usage. However, another limitation was not having a mechanism to automatically download extensive app usage data directly from all of the participants due to budget constraints; thus, we had to rely on manual downloads of app usage data among the participants who completed follow-up. Our analyses indicated that participants receiving SNAP benefits were more likely to complete follow-up, but we did not find other demographic differences between completers and noncompleters. However, it is possible that the attrition of noncompleters could have introduced bias in the app usage data. Budget limitations prevented us from continuously modifying and upgrading the app prototype based on ongoing feedback while it was being beta-tested. We plan to incorporate automatically synced app analytics and additional iterative design cycles to develop the next version of the app. Finally, as a pilot study of a prototype app, our sample size was relatively small, particularly for the app usage logs that could not be collected on all participants. However, the findings have generated very useful insights that will inform our future work with this app on a larger scale.

### Conclusions

Our team plans to leverage the findings and lessons learned from the CHEW prototype development and testing process to guide our next phase of research, in which we will undergo a new phase of iterative development and usability testing to upgrade to CHEW version 2.0. We plan to create both Android and iOS versions that will be adapted to communicate directly with Tennessee’s forthcoming EBT system, overcoming many of the technical challenges we faced in the prototype phase associated with the paper voucher system, multiple participants per family, and special dietary needs. Subsequently we will assess the impact of the app on improving food purchasing behaviors, parent feeding strategies, and child dietary intake in a randomized trial. We will also explore how certain educational components of the app could be adapted for a Web-based program and print resources to increase accessibility beyond those who do not use smartphones.

With further development, the CHEW app offers the potential to be disseminated eventually to WIC programs in states across the country to improve nutrition and reduce obesity risk among preschoolers. Future versions of the CHEW app will need an efficient mechanism to update the app with periodic revisions to the WIC food packages, changes to the WIC-approved food lists, and changes in the inventory of WIC-authorized products from a wide range of vendors. Since WIC is a federal program that is administered by the states and includes several options for states to customize the program, considerable variation exists across states regarding which foods are approved. Thus, in order to disseminate the app to states beyond Tennessee, future iterations would need to be adapted to specific state-level rules and approved food databases.

The WIC program represents an ideal setting to disseminate technology-based behavioral interventions targeting parents of young children when early intervention is needed to reduce the risk of childhood obesity. Parents and the home environment play a crucial role in primary prevention of childhood obesity [41-43]. Smartphone apps offer the potential to make a substantial impact on population health outcomes, even if the magnitude of impact on each individual is relatively small, given the potential for smartphone apps to reach large numbers of people [44]. More applied research is needed to develop and evaluate innovative interventions implemented in the WIC context such as the CHEW app.

## Abbreviations

CAB: Community Advisory Board
CHEW: Children Eating Well
CVV: cash value voucher
EBT: electronic benefits transfer
NIH: National Institutes of Health
SNAP: Supplemental Nutrition Assistance Program
UPC: universal product code
USDA: US Department of Agriculture
WIC: Special Supplemental Nutrition Program for Women, Infants, and Children
